# Total Iron Concentrations in Different Biological Matrices—Influence of Physical Training

**DOI:** 10.3390/nu14173549

**Published:** 2022-08-28

**Authors:** Francisco J. Grijota, Víctor Toro-Román, Jesús Siquier-Coll, María C Robles-Gil, Diego Muñoz, Marcos Maynar-Mariño

**Affiliations:** 1 Faculty of Life and Nature Sciences, University of Nebrija, Campus La Berzosa, Calle del Hostal, 28248 Hoyo de Manzanares, Madrid, Spain; 2 Faculty of Sport Sciences, University of Extremadura, Avenida de la Universidad s/n, 10003 Cáceres, Extremadura, Spain; 3 SER Research Group, Center of Higher Education Alberta Giménez, Comillas Pontifical University, Costa de Saragossa 16, 07013 Palma Mallorca, Islas Baleares, Spain

**Keywords:** iron, platelets, erythrocytes, plasma, serum and urine

## Abstract

Iron (Fe) is one of the most widely studied trace mineral elements. Fe metabolism and homeostasis could be altered by physical training. The aim of this study was to analyze the influence of long-term physical training on serum, plasma, urine (extracellular), erythrocyte and platelet (intracellular) Fe concentrations. Forty men from the same geographical area divided into a training group (TG; *n* = 20; 18.15 ± 0.27 years) and a control group (CG; *n* = 20; 19.25 ± 0.39 years) participated in this study. The TG was composed of soccer players of the highest youth category. The CG consisted of young people who did not follow any training routine and had not practiced any sport for at least the previous six months. The TG showed higher plasma and serum Fe concentrations (*p* < 0.05), but lower concentrations in erythrocytes and platelets compared to the CG (*p* < 0.01). Due to the differences observed in the extracellular and intracellular compartments, it seems necessary to perform a global Fe analysis to assess Fe status.

## 1. Introduction

Iron (Fe) is one of the most abundant elements in the earth’s crust and one of the most studied micronutrients [1]. It is a biologically essential component of all living organisms and plays a key role in exercise-related processes [2,3]. Fe is mainly found in the body in complex forms bound to proteins (hemoproteins) such as heme compounds (hemoglobin or myoglobin), heme enzymes (cytochromes) or non-heme compounds (ferritin). Furthermore, Fe could be linked with Sulphur (Fe-S), forming proteins with oxidoreductive activity [4].

Body Fe content is approximately 3.5 g in a 70 kg individual. Fe is mainly distributed within hemoglobin (about 65% of the total) [5] in the human body, approximately 10% of Fe is present in muscle fibers within myoglobin, enzymes and cytochromes [5]; the remaining body Fe is stored in the liver, macrophages and bone marrow [6]. Once absorbed, Fe is transported by transferrin to cells or bone marrow for erythropoiesis [1]. Within the cell, Fe can be stored in the cytosol as ferritin and hemosiderin. Dietary Fe is found in heme (10%) and non-heme (90%) forms. Non-heme dietary Fe is mainly in a ferric form (Fe^3+^) that is not bioavailable and must be reduced to the ferrous form (Fe^2+^) [7]. Heme Fe is absorbed at a higher rate than the non-heme form by enterocytes [5].

Fe is vital in the transportation of oxygen, as a component of hemoglobin and myoglobin [8]. In addition, the Fe-S complex is involved in enzymes responsible for electron transport, energy generation in mitochondrial respiration, the Krebs cycle and ribonucleotide reductase, which is essential for DNA synthesis. Fe plays important roles in hormone synthesis, muscle division and growth [2,5,9].

Fe metabolism and homeostasis could be altered by exercise and nutrition [10,11,12]. Physical exercise could impair Fe status through several mechanisms, including exercise-induced inflammation, hemolysis, or losses through sweat or urine [13,14,15]. Elsewhere, Fe deficiency is one of the most prevalent nutritional deficiencies in the general population [16,17]. In athletes, Fe deficiency is more prevalent, most commonly affecting women [18,19,20]. Fe status could influence physiological function and performance. Fe deficiency impairs aerobic capacity, reduces muscle function and leads to increased lactate concentrations [21].

Soccer is characterized by intermittent work periods combining low-, moderate- and high-intensity running, constantly interrupted by jumping, dribbling, tackling, breaking and heading. These physiological demands can compromise the musculoskeletal, nervous, immune and metabolic systems, which could be reflected in changes in biochemical and hematological parameters [22].

Previous authors reported no differences between male soccer players and control subjects in parameters such as serum Fe, ferritin or transferrin saturation [23]. However, when comparisons are made throughout the sports season on indicators of Fe status, significant changes are observed in hematocrit, ferritin, mean corpuscular hemoglobin and mean corpuscular volume in various assessments [24].

As previously noted, evaluation of Fe status is usually performed indirectly using several indicators [25]. These include: serum ferritin, transferrin saturation and hemoglobin concentration, which are the most commonly used parameters to assess Fe status indirectly [26,27,28,29]. However, some indicators such as ferritin or transferrin saturation could be altered by inflammation or diurnal fluctuations [30,31]. Previously, Sherwood et al. [32] reported that serum, urine or tissue Fe concentration assessments are accurate when dealing with disorders of Fe metabolism. Normally, Fe levels are assessed in extracellular compartments such as serum or plasma [13,26,33]. Nevertheless, studies analyzing Fe concentrations in intracellular compartments are scarce [10,34]. Assessments of Fe status in athletes are usually performed in one or two compartments [13,26,33], with limited studies with more than two compartments [34,35]. Regarding intracellular Fe determinations, these are usually assessed in erythrocytes [10,34]. Nonetheless, the study of erythrocyte Fe can be time-consuming because the half-life of erythrocytes is about 120 days [36]. The study of platelets could be a reliable and current alternative due to their reduced half-life (approximately 10 days) [37]. Relationships between Fe status and platelet parameters have been reported [38].

It has recently been reported that deficits in intracellular compartments could exist despite there being no differences in the extracellular compartments of trace mineral elements (TME) [39,40,41]. Therefore, a complete assessment of several compartments must be performed simultaneously when assessing TME status. Hence, the aim of this study was to analyze the influence of long-term physical training on serum, plasma, urine (extracellular), erythrocyte and platelet (intracellular) Fe concentrations simultaneously with a method not used until now: inductively coupled plasma mass spectrometry (ICP-MS).

## 2. Materials and Methods

The participants, materials and methods were previously described by Toro-Román et al. [39,41]. However, they are explained below. TG reduced the intensity and volume of training the two previous days and rested the day before the evaluations to avoid fatigue. 

### 2.1. Subjects

Forty healthy men divided into a training group (TG; *n* = 20; 18.15 ± 0.27 years) and a control group (CG; *n* = 20; 19.25 ± 0.39 years) voluntarily participated in this study. All of them were informed of the purpose of the study and signed an informed consent form. The protocol was reviewed and approved by the Biomedical Ethics Committee of the University of Extremadura (Cáceres, Spain) following the guidelines of the Helsinki ethical declaration (code 13/2021). All study participants had resided in the area of Cáceres (Spain) in the previous 24 months prior to the start of the study.

Inclusion criteria were the following: to be a man, not to follow any special diet or take vitamin/mineral supplements, or specific supplementation, medication or over-the-counter medication and not to have had any injuries or illness during the investigation or at least 6 months before the study.

The TG consisted of 20 semi-professional soccer players from a national youth division of honor team. All of them had training experience of at least 5 years and performed regular soccer training of approximately 10 h per week. The CG was composed of 20 youngsters who did not follow any physical training plan and had not practiced any sport in the previous 6 months. A physical activity questionnaire—short form (IPAQSF) Spanish version was used to assess the levels of physical activity [42,43].

To ascertain Fe intake, all participants completed the nutritional survey. The survey consisted of a 4-day daily nutritional record for three working days and one weekend. Participants indicated the frequency and amount of each food consumed, and the nutritional composition was assessed using food composition tables [44].

The evaluations were carried out at the end of the first month of the season. The characteristics of the training sessions performed by TG are shown in Table 1.

### 2.2. Anthropometric Measurements

The anthropometric measurements obtained were height, weight, skinfolds (abdominal, suprailiac, subscapular, tricipital, thigh and leg), bone diameters (bistyloid, humeral biepicondyle and femoral biepicondyle) and muscle perimeters (relaxed arm and leg). Body height was measured using a wall-mounted stadiometer (Seca 220. Hamburg, Germany). Body weight was measured using calibrated electronic digital scales (Seca 769. Hamburg, Germany) in nude, barefoot conditions. A Holtain© 610ND (Holtain, Crymych, UK) skinfold compass, a Holtain© 604 (Holtain, Crymych, UK) bone diameter compass, and a Seca© 201 (Seca, Hamburg, Germany) brand tape measure, were used for the anthropometric assessments. Anthropometric measurements were taken by the same operator. The guidelines of the Spanish Group of Kinanthropometry were used to calculate the muscle and fat percentages [45].

### 2.3. Sample Collection and Fe Determination

A sample of 12 mL of venous blood was withdrawn from each subject using a plastic syringe fitted with a steel-free catheter to avoid Fe contamination after a fasting period of at least 8 h. First, 2 mL of blood were used for the determination of hematological parameters using an automatic cell counter (Coulter Electronics LTD, Model CPA; Northwell Drive, Luton, UK).

For serum, a blood sample of 5 mL was collected in a metal-free polypropylene tube and then centrifuged at 3000 rpm for 15 min. Serum was aliquoted into an Eppendorf tube and was left to stand at −80 °C.

For plasma, a blood sample of 5 mL was collected in a metal-free polypropylene tube with ethylenediaminetetraacetic acid (EDTA) and then centrifuged at 1800 rpm for 8 min. The platelet-rich plasma obtained was collected in a metal-free polypropylene tube and centrifuged for 15 min at 3000 rpm and the plasma was aliquoted into an Eppendorf tube and was left to stand at −80 °C. One milliliter of pure water was added to the tube of the concentrate of platelets and stored at −80 °C.

The erythrocytes were extracted from the rest of the blood and were washed with 0.9% sodium chloride (NaCl) three times. They were then aliquoted into Eppendorf tubes and conserved at −80 °C.

The first urine sample was obtained from all subjects, collected in polyethylene tubes previously washed with diluted nitric acid, and frozen at −80 °C until analysis. 

Techniques for Fe determination in plasma, serum, urine, erythrocytes and platelets were similar to those reported by Toro-Román et al. [39,40,41].

Fe determination was performed by ICP-MS (7900; Agilent Tech., California, CA, USA). The instrument had a fast dual simultaneous mass detector, a high-frequency hyperbolic quadrupole, and a fourth-generation reaction octopole system that allowed operation in two modes: no reaction gas and kinetic energy discrimination with helium as the collision gas. The collision gas and the argon for the plasma were 99.999% pure and supplied by Praxair (Madrid, Spain). In addition, it had a 27 MHz variable frequency generator, cooled spray chamber, low-flow sample introduction system, Off-Axis ionic lenses, and an extraction interface with high transmission and matrix tolerance.

For plasma, serum, and urine samples, the reagents used were 69% nitric acid (TraceSELECT™, Fluka™) and ultrapure water obtained from a Milli-Q system (Millipore^®^, Burlington, MA, USA). A 400 µgL-1 Rhodium dilution was used as an internal standard and continuously fed into the apparatus with the aid of the three-channel peristaltic pump. The 0.2 mL of the samples to a volume of 5 mL with a 1% nitric acid solution prepared from a 69% commercial one. The equipment was calibrated with several calibration standards prepared from commercial multielemental solutions of certified standards.

For erythrocyte and platelet samples, the reagents used in method development and sample analysis were 69% nitric acid, hydrogen peroxide (TraceSELECT™, Fluka™) and ultrapure water obtained from a Milli-Q system (Millipore^®^, Burlington, MA, USA). A 400 µgL-1 Ytrium and Rhodium dilution was used as internal standard, which was continuously fed into the apparatus with the aid of the three-channel peristaltic pump.

Samples were weighed on a precision balance ± 0.4 g and introduced into glass tubes for microwave digestion. Then, 3.5 mL of a 3:1 mixture of 69% nitric acid and hydrogen peroxide (TraceSELECT™, Fluka™) was added.

Once digested, the resulting solutions were diluted to 25 mL with MilliQ water. The limits of detection and limits of quantification for Fe were 0.17 μg/L and 1.69 μg/L. The linearity of the calibration curves in plasma, serum, urine, erythrocytes and platelets were greater than 0.985. The values of the standard materials for this element (10 μg/L) used for quality controls coincided with intra- and inter-assay coefficients of variation lower than 5%.

### 2.4. Statistical Evaluations

The normality of variables was analyzed using the Shapiro–Wilk test and the homogeneity of the variances with the Levene test. Student’s t-test was used to compare the different parameters of both groups. A *p* < 0.05 was considered statistically significant. Effect size (ES) was calculated [46]. ES of 0.2, 0.4, and 0.8 were considered small, moderate, and large, respectively [47]. Statistical analyses were carried out with IBM SPSS Statistics 22.0 for Windows (SPSS Inc., Chicago, IL, USA). 

### 2.5. Incremental Test until Exhaustion

Participants performed a maximal incremental test to exhaustion to assess cardiorespiratory fitness. The protocol started at 8 km/h for CG and 10 km/h for TG. Every minute it increased by 1 km/h with a 1% slope. All the participants performed a 15-minute warm-up by running progressively until they achieved the starting speed of the test. A treadmill (Trac Alpin 4000, ERGOFIT, Pirmasens, Germany), equipped with a gas analyzer (Geratherm Respiratory GMBH, Ergostik, Ref 40.400, Corp Bad Kissingen, Geschwenda, Germany) and a Polar pulsometer (Polar^®^ H10, Kempele, Finland) were used.

## 3. Results

Table 2 reflects the characteristics of the participants. The TG recorded lower weight, fat percentage and resting heart rate compared to the CG (*p* < 0.05). However, the TG had higher muscle percentage, maximal oxygen consumption (VO2max), expiratory volume (VE) and physical activity levels (*p* < 0.05).

Table 3 displays the energy, macronutrient and Fe intakes of the study participants. There were no differences between groups.

Table 4 shows the hematological parameters in both groups. No significant differences were observed among participants.

Table 5 reports plasma, serum and urine Fe concentrations. The TG showed higher plasma and serum Fe concentrations (*p* < 0.05).

Table 6 illustrates the erythrocyte and platelet Fe concentrations. The TG had lower erythrocyte and platelet Fe values, both in absolute and relative terms compared to the CG (*p* < 0.01).

## 4. Discussion

This research aimed to analyze the influence of regular physical training on intracellular and extracellular Fe concentrations using a highly reliable technique for this purpose such as ICP-MS. The TG showed elevated serum and plasma Fe concentrations compared to the CG (*p* < 0.05). However, the TG had lower concentrations in erythrocytes and platelets, both in absolute and relative values (*p* < 0.01). Soccer players exposed to a demanding calendar during a competitive period may be predisposed to a Fe deficiency that could compromise their performance and metabolic health towards the end of the season [48]. Accumulated fatigue and inadequate recovery time during a competitive period may predispose players to alterations in the state of Fe [20].

Fe status has been associated with athletic performance [25,49,50]. Indirect assessment of Fe through other markers could have certain limitations in populations such as athletes [51]. Ferritin levels could only indicate the magnitude of Fe stores and not its functional reserve (amount of Fe in hemoglobin, myoglobin and other enzymes) [52]. Detection of Fe deficiency based on ferritin assessment is limited in athletes since physical training could induce inflammatory responses, especially in acute phases [52]. It has been reported that increases in plasma ferritin could be maintained for several days after strenuous physical exercise [52,53]. Elsewhere, hemoglobin assessment to analyze Fe status has certain limitations because a low hemoglobin level could be due to an expansion of plasma volume [54]. Regarding serum Fe evaluation, it is known to have a high diurnal variability. Diurnal Fe values are higher compared to values obtained in the afternoon, which may not be a reliable measure of Fe status [55]. Therefore, due to the different limitations of the markers of Fe status, a multicompartmental total Fe analysis seems to be necessary for a more complete assessment [39]. The Fe concentrations obtained in each compartment, by ICP-MS, were within the ranges reported in other investigations with similar techniques [10,56,57,58].

Fe is the most abundant trace element involved in cell metabolism and growth of organisms [59]. Regular assessment of Fe status in athletes is crucial for optimal performance, especially in endurance sports. Physical performance could decrease, leading to constant fatigue and cognitive impairment when Fe stores are inadequate [10]. The body has no mechanism to restore Fe losses due to physical exercise. Therefore, adequate dietary intake is essential for athletes during periods of intense training [60]. As mentioned above, Fe deficiency is one of the most common deficits in the general population and among athletes, mostly in women [61]. In the present study, Fe intake in both groups was higher than dietary reference intakes (DRI) (9–11 mg/day) [62]. Nutritional intake is the first step in correcting Fe deficiency. Heme Fe uptake and absorption is higher compared to free or non-heme Fe [61]. Moreover, hepcidin, a hormone that regulates Fe availability, plays an important role in Fe absorption [63]. This hormone is increased, among other factors, by inflammation, decreasing Fe absorption [14]. The relationship between exercise, inflammation and hepcidin activity has been well studied because exercise is a potent inflammatory stimulus [14,64,65]. Furthermore, low energy availability resulting from reduced energy intake and/or increased training load is associated with an increase in hepcidin, triggering abnormal upregulation and impairment of Fe metabolism [11]. Therefore, the timing of Fe intake and energy availability are two factors that must be taken into account when optimizing Fe status.

Concerning extracellular Fe concentrations, the TG showed higher serum and plasma Fe concentrations compared to the CG (*p* < 0.05). The Fe values in the TG were higher than those reported in other studies in soccer players [24,66]. Rakhra et al. [33] and Mettler and Zimmermann [67] observed increased plasma Fe values in active people and marathon runners with respect to the results obtained in this study. However, other authors observed no differences with the CG and other sports disciplines [21,68,69], or even reported reduced values in athletes [70]. Regarding serum concentrations, Schumacher et al. [71] reported increased Fe values in international endurance athletes compared to local athletes. Similarly, Constantini et al. [72] reported higher Fe levels in swimmers and racquet sports athletes compared to gymnasts. Nevertheless, other authors observed no differences in plasma Fe concentration over the season in soccer players [24,66] and endurance runners [73]. In contrast, Ponorac et al. [26] and Sandström et al. [70] documented lower Fe concentrations in women athletes compared to the CG.

The differences in plasma and serum Fe concentrations between the groups could be multifactorial. Hemolysis occurring during training, specifically in soccer, could be an important factor [74,75]. Another factor may be hemoconcentration due to changes in plasma volume caused by regular training [12,75,76]. Soccer is an anaerobic-aerobic sport where sprints, acceleration, deceleration, changes of direction and trauma are prevalent [77]. These actions could enhance muscle damage and hemolysis [74]. Elevated body temperature, metabolic acidosis and hemoconcentration, observed during physical exercise, reduce the osmotic resistance of erythrocytes [78]. Previous studies suggested that the intravascular hemolysis observed during exercise is a consequence of injury to older erythrocytes, which are less elastic and more susceptible to damage [79]. The same authors documented an inverse correlation between hemolysis and levels of the erythrocyte membrane protein spectrin [79]. These results could support the hypothesis that structural alterations of erythrocyte membranes increase the susceptibility of these cells to hemolysis, leading to elevated plasma levels of free Fe. Regarding plasma volume, it is necessary to consider the change in plasma volume during exercise when assessing the effects of exercise on the concentrations of non-diffusible blood components [80]. As blood components with high molecular weight cannot freely cross vascular walls, their serum concentrations may increase or decrease according to changes in plasma volume and vascular tone [81].

Regarding intracellular concentrations, lower values were observed in the TG compared to the CG in erythrocytes and platelets (*p* < 0.01). It is unusual to assess Fe in these compartments. Similar results were reported by Maynar et al. [10] in erythrocytes. In addition, previous authors observed an inverse relationship (r = −0.744; *p* = 0.000) between training status and the amount of Fe in this compartment. Concerning platelets, inverse relationships between serum Fe levels and platelet and plateletcrit numbers have been found [82]. However, to our knowledge, there is no information on platelet Fe content in athletes.

Erythrocyte Fe deficiencies could be due to insufficient Fe intake in the months prior to the study. As mentioned above, the half-life of erythrocytes is approximately 120 days [36]. Additionally, the intracellular concentration of TME in erythrocytes is not affected by the acute inflammatory response or by short-term diets [83]. Therefore, the reported data on erythrocyte Fe concentrations may not be recent. On the other hand, it could be related to the hemolysis mentioned above. It is known that physical exercise, characterized by a significant strain on the athlete, especially in competitive situations, leads to a more rapid aging of erythrocytes. The half-life of erythrocytes in athletes could be significantly shorter than in non-exercising subjects [84]. Exercise-induced hemolysis could be implicated in the suboptimal erythrocyte Fe status of athletes [85]. This could trigger Fe loss as a consequence of erythrocyte membrane destruction and subsequent release of hemoglobin and Fe to extracellular compartments [60]. The rate of erythrocyte destruction could be altered as a consequence of repetitive training [76]. Elevated red cell turnover could be a consequence of blood loss or hemolysis during training [76]. Related to the above, regular physical exercise causes stressful physiological situations, such as increased oxidative stress, which can alter the membrane properties of red blood cells and trigger eryptosis [86]. As with programmed nucleated cell death or apoptosis, eryptosis is a coordinated suicidal death that ultimately eliminates defective cells without cell membrane rupture and release of intracellular material. It is considered a valuable mechanism to avoid a complication of hemolysis by initiating a program of cell death with controlled elimination before any damage can cause uncontrolled hemolysis [87]. Iron-deficient erythrocytes are known to be more sensitive to eryptosis [88]. Therefore, erythrocyte iron concentrations are essential in promoting eryptosis and reducing hemolysis complications.

Regarding platelet Fe differences, these could be due to insufficient current Fe intake, since platelets provide more current information due to their short half-life (approximately 2 weeks). Short-term variations in the status of other TMEs only seem to influence newly synthesized cells, as the incorporation of TMEs occurs in bone marrow cells [89]. Further research on Fe concentrations in this compartment is needed in order to provide more current information.

The present study has some limitations: (a) the small sample size; (b) the absence of complementary parameters of Fe metabolism such as ferritin or transferrin; (c) the absence of plasma volume assessment; and (d) the absence of women athletes, since Fe metabolism is not only subject to changes related to physical exercise or diet, but monthly blood loss through menstruation could lead to a greater decrease.

## 5. Conclusions

Regular physical training could affect extracellular and intracellular Fe concentrations. Specifically, physical training could increase Fe concentrations in extracellular compartments (plasma and serum) and decrease intracellular Fe concentrations (erythrocytes and platelets). We encourage a global analysis of TME by assessing extracellular and intracellular compartments simultaneously due to the discrepancies in Fe determination in the present study.

## Figures and Tables

**Table 1:** Team and training characteristics of soccer players (TG).

Parameters		TG (*n* = 20)
Position (%)	Goalkeeper	10.00
	Defense	35.00
	Midfielder	40.00
	Forward	15.00
Matches played (*n*)		1.00
Trainings (*n*)		17.04 ± 4.39
Training sessions (min)		1534.28 ± 416.71
Training load (RPExmin)		2778.54 ± 1118.13

RPE: rate of perceived exertion.

**Table 2:** Characteristics of the participants.

Parameters	CG (*n* = 20)	TG (*n* = 20)
Weight (kg)	73.45 ± 9.04	68.59 ± 4.18 *
Height (m)	1.79 ± 0.06	1.76 ± 0.04
Muscle Mass (%)	44.22 ± 5.71	49.03 ± 2.56 *
Fat Mass (%)	15.64 ± 5.78	9.32 ± 2.76 *
VO_2_ max (mL/Kg/min)	45.61 ± 4.95	61.02 ± 4.35 **
VE (L/min)	88.34 ± 11.18	120.56 ± 18.79 **
Resting HR (bpm)	67.31 ± 6.49	54.41 ± 5.29 *
Maximum HR (bpm)	189.3 ± 7.1	193.8 ± 6.5
Physical activity (MET-hours/weekly)	27.36 ± 4.45	56.13 ± 6.21 **

CG = control group; TG = training group; VO_2max_ = maximal oxygen consumption; VE = expiratory volume; HR = heart rate; MET = metabolic equivalent of task; * *p* < 0.05; ** *p* < 0.01.

**Table 3:** Nutritional intake of participants.

Parameters	CG (*n* = 20)	TG (*n* = 20)
Energy (kcal/day)	2112.34 ± 345.78	2456.16 ± 504.11
Water (L/day)	1.145 ± 0.241	1.421 ± 0.356
Carbohydrates (g/kg/day)	3.11 ± 1.28	3.98 ± 1.78
Proteins (g/kg/day)	1.25 ± 0.37	1.44 ± 0.41
Lipids (g/kg/day)	1.51 ± 0.47	1.64 ± 0.31
Fe (mg/day)	13.67 ± 3.21	14.18 ± 2.74

CG = control group; TG = training group; Fe = Iron.

**Table 4:** Erythrocyte, hemoglobin, hematocrit and platelet values.

Parameters	CG (*n* = 20)	TG (*n* = 20)	ES
Erythrocytes (cell 10^12^/L)	4.81 ± 0.72	4.76 ± 0.89	0.14
Hemoglobin (g/dL)	14.75 ± 0.78	14.14 ± 0.95	0.08
Hematocrit (%)	43.24 ± 1.04	42.65 ± 1.23	0.10
Platelets (cell 10^9^/L)	190.23 ± 67.13	198.35 ± 60.51	0.17

CG = control group; TG = training group; ES = effect size.

**Table 5:** Fe concentration in extracellular compartments.

Parameters	CG (*n* = 20)	TG (*n* = 20)	*p*	ES
Plasma (μg/L)	2023.37 ± 514.61	2486.51 ± 573.24	0.016	0.61
Serum (μg/L)	1536.13 ± 416.29	1840.00 ± 583.64	0.031	0.43
Urine (μg/L)	3.77 ± 0.99	3.62 ± 1.61	0.641	0.02

CG = control group; TG = training group; ES = effect size.

**Table 6:** Fe concentration in intracellular compartments.

Parameters	CG (*n* = 20)	TG (*n* = 20)	*p*	ES
Erythrocytes (mg/L)	927.38 ± 115.88	787.05 ± 85.71	<0.001	1.00
Erythrocytes (μg/cell10^−6^)	193.21 ± 21.06	167.03 ± 17.65	0.003	0.84
Platelets (μg/L)	54.04 ± 36.79	25.34 ± 9.44	0.008	0.69
Platelets (pg/cell 10^−3^)	0.284 ± 0.164	0.127 ± 0.082	0.006	0.73

CG = control group; TG = training group; ES = effect size.

## Data Availability

Not applicable.
